# Analysis of the relationship between GLUT family in the progression and immune infiltration of head and neck squamous carcinoma

**DOI:** 10.1186/s13000-023-01377-x

**Published:** 2023-08-04

**Authors:** Bing Li

**Affiliations:** Department of Clinical Laboratory, Nanjing Stomatological Hospital, Affiliated Hospital of Medical School, Nanjing University, No 30 Zhongyang Road, Nanjing, 210008 China

**Keywords:** Head and neck squamous cell carcinoma, Biomarker, Prognosis, Glucose transporter type family, Bioinformatics analysis

## Abstract

Head and neck squamous cell carcinoma (HNSCC) causes much health and economic burden, and the therapeutic results must be improved. Glucose metabolism is an essential component of tumor metabolism and is instrumental in its development. Glucose transporter types (GLUTs) can uptake glucose from the extracellular matrix (ECM), regulating cellular metabolism in several cancers. However, the function of different GLUT proteins in HNSCC remains unclear. To clarify the role of GLUTs in HNSCC, several open-access online databases (Oncomine, GEPIA, Kaplan–Meier, cBioPortal, GeneMANIA, and TIMER) were used to evaluate the differential expression, clinical significance, genetic alteration, and relative immune cell infiltration. The expression of GLUTs was detected in clinical patient samples by immunohistochemistry. The mRNA level of SLC2A1/3 significantly increased in HNSCC, while SLC2A4 reduced. SLC2A3 was related to the advanced clinical stage and short overall survival (OS) in HNSCC. Also, higher SLC2A1/2 mRNA expression was related to shorter OS in HNSCC patients. The expression of GLUTs was related to diverse immune cells, including B cells, CD4^+^ T cells, CD8^+^ T cells, dendritic cells (DCs), macrophages, and Treg cells in HNSCC. Moreover, the high expression of GLUTs was demonstrated by immunohistochemistry in patient tissues. GLUTs might have a potential role in HNSCC's progression and development. Therefore, the current findings might offer a novel perception for selecting GLUT family prognostic markers and treatment for HNSCC patients.

**Supplementary Information:**

The online version contains supplementary material available at 10.1186/s13000-023-01377-x.

## Impact statement

Herein, we provided evidence for the relationship between GLUTs and HNSCC using bioinformatics approaches. The expression of GLUTs was significantly associated with the clinical cancer stage and short overall survival (OS) in HNSCC patients. Also, the expression of GLUTs was significantly correlated with the infiltration of diverse immune cells. We also conducted immunohistochemistry to test the expression of GLUTs in patient tissues. Our findings might provide novel insights for selecting GLUT family prognostic markers in HNSCC. These findings might also help develop new personalized treatments for HNSCC by monitoring the immune microenvironment, which might play a significant role in devising personalized therapeutic approaches for HNSCC patients.

## Introduction

Head and neck squamous cell carcinoma (HNSCC) causes much health and economic burden, accounting for 3% of all malignancies [1, 2]. HNSCC can occur on several sites, mainly in the oral cavity, larynx, and hypopharynx. It is associated with lifestyle risk factors, including the most common alcohol and tobacco [3–5]. The surgery section is the most common therapy for HNSCC. However, the dysfunctions after surgery are inconvenient to patients. Many studies have shown that metabolic reprogramming in HNSCC and glucose metabolism is critical in oncogenesis [6], the most common component of energy metabolism, and its potential therapeutic value should be explored.

As a glucose membrane transporter, the glucose transporter type (GLUT, also known as SLC2A) can uptake glucose from the extracellular matrix (ECM) and regulate cellular metabolism in several cancers [7]. Many GLUT family proteins are oncogenes involved in the progression of various tumors, such as bladder and prostate cancers [8]. The prognostic and immunotherapeutic roles of SLC2A have been explored in numerous cancers. However, studies on GLUT family proteins and their relationship are rare.

Although GLUTs can be categorized into three classes according to the gene sequence similarity and substrate specificity: Class 1 (GLUTs 1–4 and 14), Class 2 (GLUTs 5, 7, 9, and 11), and Class 3 (GLUTs 6, 8, 10, 12, and 13/HMIT), all in all, GLUTs 1–4 were the most famous and investigated members in field of cancer research [7], and play a predominant role in maintaining cellular glucose uptake and functional metabolic homeostasis during carcinogenesis. Therefore, the expression patterns and prognostic roles of these GLUT members, GLUTs 1–4, in tumors were investigated in this study. GLUT-1 expression has been associated with therapeutic resistance in multiple malignancies, including in HNSCC, owing to the effects on DNA repair [9]. But the GLUT2 was detected in both malignant and normal epithelial cells. Also, the GLUT3 was found to be highly expressed in the tumor site, compared to the healthy tissue [10]. The GLUT4 expression is quite prevalent expressed in the HNSCC, but the studies in patient is rare [11]. Hence, to analyze the connection between GLUT 1,2,3,4 and the immune infiltration of HNSCC, we used various databases (Oncomine, GEPIA, Kaplan–Meier, cBioPortal, GeneMANIA, and TIMER) and methods to determine the potential oncogenic values and immune infiltration of distinct GLUT family members in HNSCC patients. GLUTs had differential expression in tumor and healthy tissues and affected immunity cells in tumor tissues. Then, we used immunohistochemistry (IHC) to demonstrate their expression in HNSCC tissues.

## Materials and methods

### Ethics approval and consent to participate

Ethical approval for this study including tumor biopsy collection was obtained from the Research Ethics Committee of Nanjing Stomatology Hospital (No.2019NL-029(KS)) and informed consent was obtained from the patients (Written form). The study was performed in accordance with the Declaration of Helsinki.

#### Differential mRNA levels

We used publicly available data in Oncomine (www.oncomine.org) to determine the mRNA levels of GLUT (encoded as SLC2A) [12]. During the analysis, the data type was mRNA, and the cut-off *p*-value and fold change (FC) were 0.01 and 1:5, respectively, with a gene rank of 10%.

#### Gene expression analysis

To compare normal and tumor tissues and the pathological stages of HNSCC, we used Gene Expression Profiling Interactive Analysis (GEPIA) to evaluate the expression variance of GLUTs (http://gepia.cancer-pku.cn/index.html) [13]. In this case, the Student’s t-test was used.

#### Kaplan–Meier (K-M) plotter

The relationship of GLUTs expression with the survival of HNSCC patients was accessed with the K-M plotter (http://kmplot.com/analysis/) [14]. The K-M plotter web page shows information about the patients, such as the number-at-risk cases, mRNA expression levels, hazard ratios (HRs), 95% confidence intervals (CIs), and *p*-values. A *p* < 0.05 was considered statistically significant. Figures were obtained using online tools.

#### Cancer genomics analysis

The genetic change and the network analysis of the GLUT family in HNSCC were obtained from cBioPortal (www.cbioportal.org), an open-access platform to explore and visualize multidimensional cancer genomic data.

#### Predictive value of GLUTs

We used GeneMANIA to weigh the predictive value of GLUTs (http://www.genemania.org) [15].

#### Protein–protein interaction (PPI) network

Also, we applied STRING (https://string-db.org/) to construct a PPI network to integrate the distinct expressions of GLUTs and their possible interactions [16].

#### Immune cell infiltration analysis

GLUTs were selected as inputs in the “Gene module,” and scatterplots were generated to visualize the correlation of GLUT expression with immune infiltration levels in HNSCC using TIMER (https://cistrome.shinyapps.io/timer/) [17].

#### IHC analysis

Ethical approval for this study, including tumor tissue collection and patients informed consent was obtained in accordance with the guidelines and policies of the Research Ethics Committee of Nanjing Stomatology Hospital. The tissue samples from the patients with HNSCC patients were used retrospectively. We collected formalin‑fixed paraffin‑embedded tissue (FFPE) specimens from patients with HNSCC patients to assess the expression of GLUTs. The IHC analysis was performed using routine procedures. First, formalin-fixed, paraffin-embedded tumor samples (4 µm) were deparaffinized. After incubation with primary antibodies (4 °C, overnight), samples were revealed using a liquid diaminobenzidine substrate chromogen system, followed by the hematoxylin staining. Finally, samples were observed.

## Results

### Differential expression of GLUTs in HNSCC patients

**Fig. 1:**
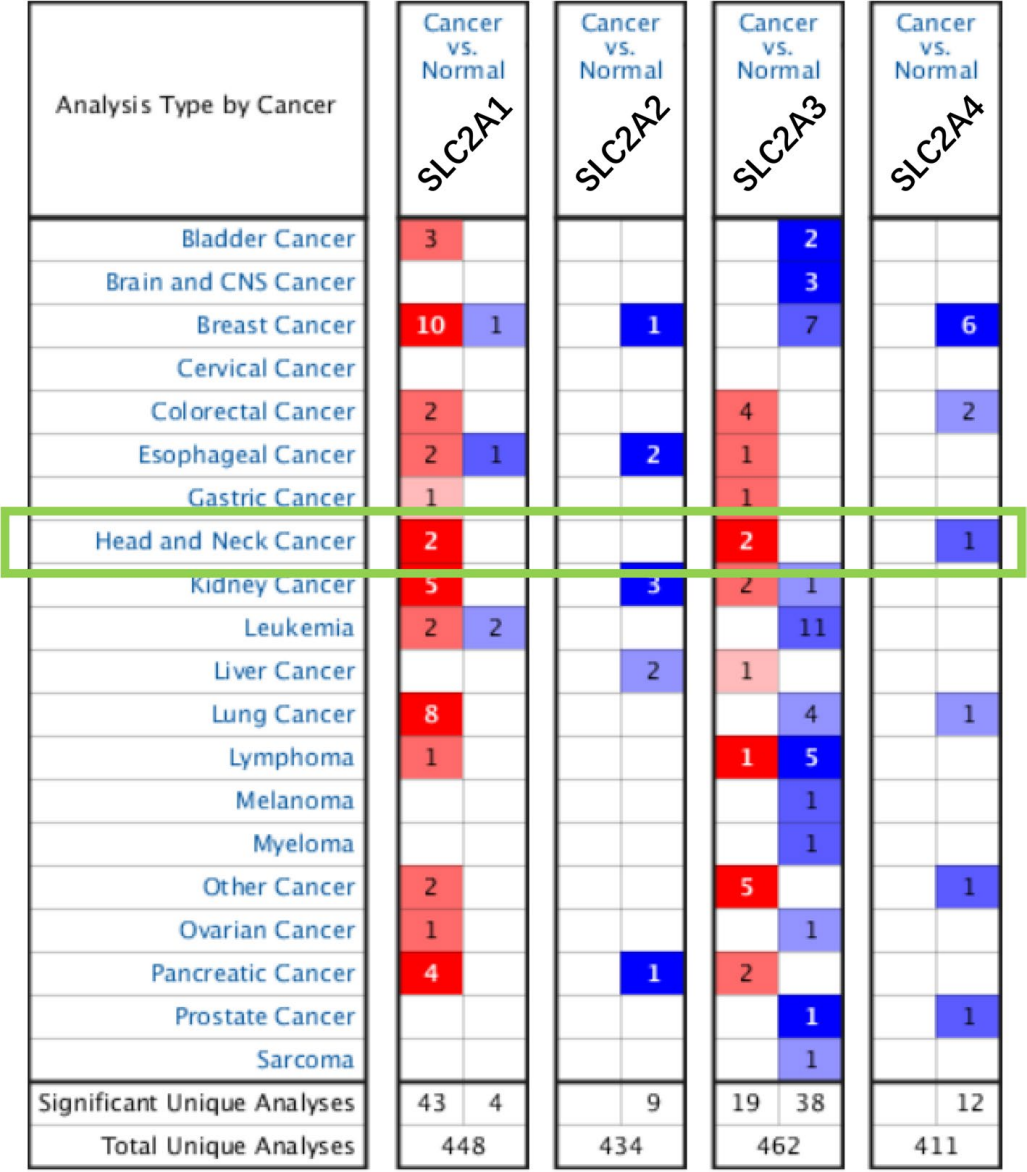
Different mRNA levels of the SLA2C family in various cancer types

First, we used the Oncomine database to explore the differential mRNA levels of the GLUT family in HNSCC patients (Fig. 1). GLUT (or SLC2A) was differentially expressed between tumor and normal tissues. SLC2A1 and SLC2A3 were remarkably elevated in HNSCC tissue, while SLC2A4 levels decreased. For instance, SLC2A1 was overexpressed in tongue squamous cell carcinoma compared to normal tissue, with an FC of 4.612 (*p* = 8.20E-8). SLC2A3 was also significantly increased in HNSCC tissues compared to normal tissues with FC of 16.709 (*p* = 2.39E-23) (Supplementary Table 1).

**Fig. 2:**
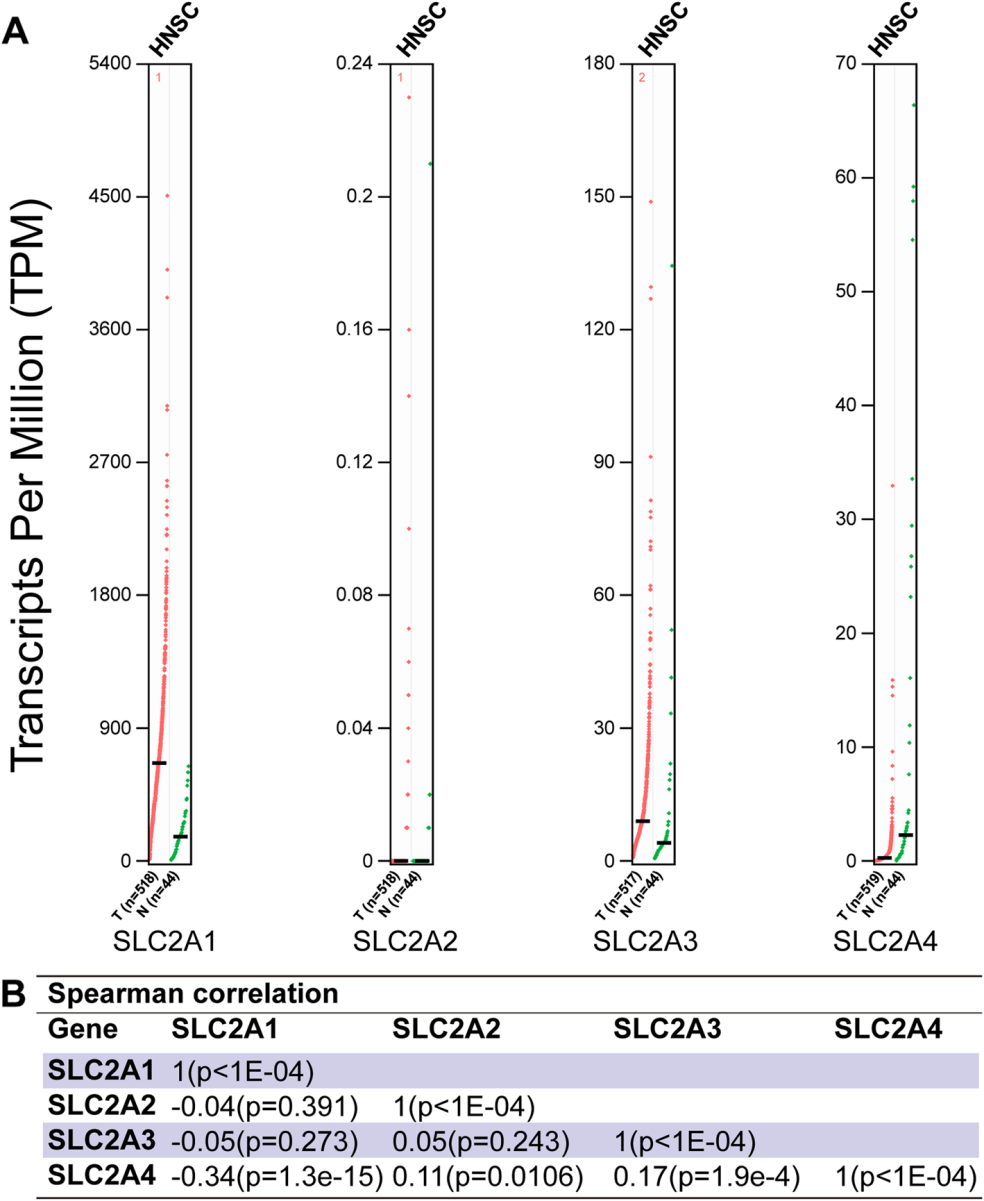
Differential expression of SLC2As in HNSCC from GEPIA (**a**), and correlations between SLC2A1-4 in HNSCC tissue samples from cBioportal (**b**)

Then, we evaluated the mRNA levels of GLUTs in HNSCC and normal tissues using GEPIA. SLC2A1 and SLC2A3 increased in HNSCC tissues, while SLC2A4 decreased (Fig. 2), consistent with the Oncomine results. The Spearman analysis showed that the expression of SLC2A4 was positively correlated with SLC2A3 but negatively correlated with SLC2A1.

**Fig. 3:**
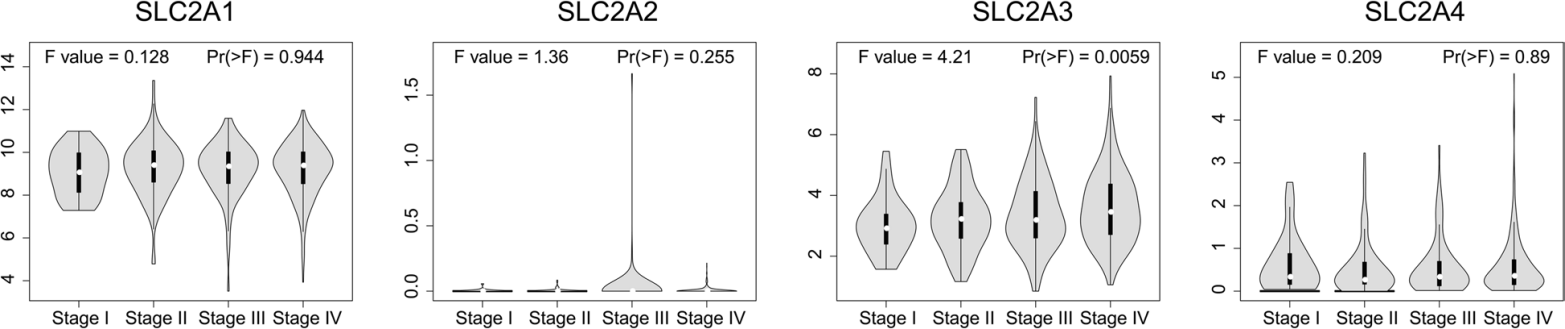
Relationship between SLC2A family and pathological tumor stage in HNSCC patients from GEPIA

Next, the relationship between GLUTs and the pathological development stage of HNSCC patients was further analyzed. SLC2A3 was highly variable (*p* = 0.0059), while the other groups did not differ (Fig. 3). These results suggested that SLC2A3 (GLUT3) might be critical in HNSCC development.

### Relationship between overall survival (OS) and recurrence-free survival (RFS) and GLUTs expression in HNSCC

**Fig. 4:**
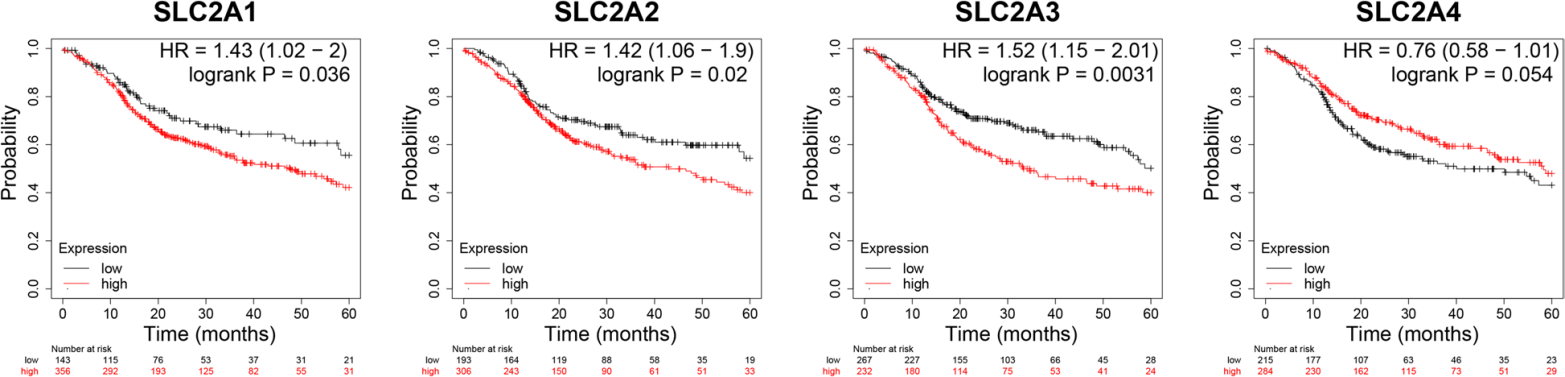
The OS prognostic value of mRNA levels of distinct SLC2A family members in HNSCC (Kaplan–Meier plotter)

To assess the value of differentially expressed GLUTs in the development and progress of HNSCC, we analyzed the relations between GLUTs and clinical results using GEPIA. The OS curves are presented in Fig. 4. HNSCC patients with high GLUT3 levels (*p* = 0.0031) were significantly associated with short OS. The increase of the GLUT2 was also associated with poor OS (*p* = 0.02), while high GLUT4 levels may be associated with long OS, but not significantly (*p* = 0.054).

**Fig. 5:**
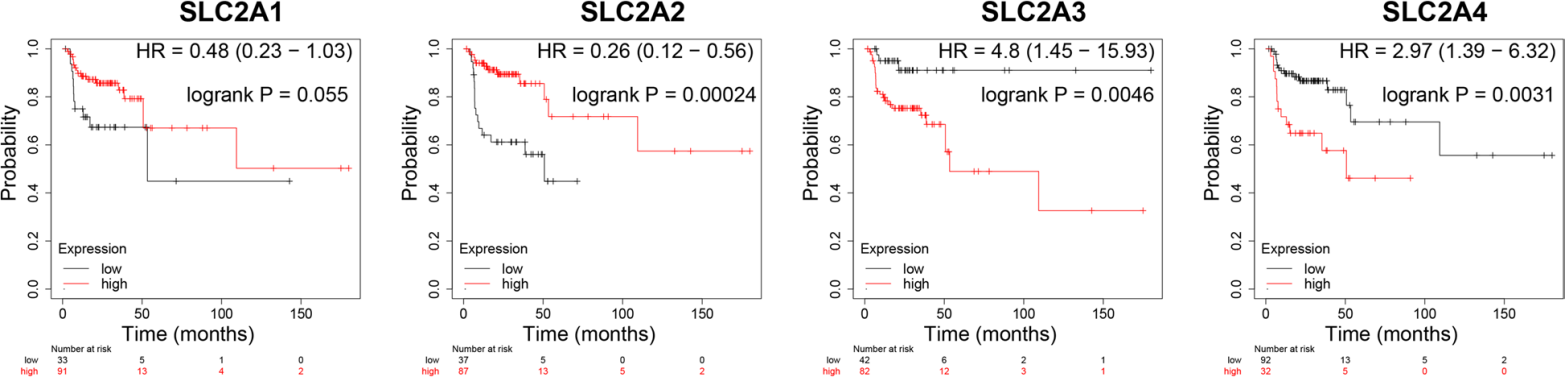
The RFS prognostic value of mRNA levels of distinct SLC2A family members in HNSCC (Kaplan–Meier plotter)

Furthermore, we determined the RFS of HNSCC patients. Unexpectedly, patients with high SCL2A3 and SLC2A4 expression presented decreased RFS, while patients with high SLC2A2 showed increased RFS (Fig. 5). Hence, the prognostic value of SCL2A2 and SCL2A4 for OS was the opposite for RFS.

### Genetic alteration, expression, and interaction of GLUTs in HNSCC patients

**Fig. 6:**
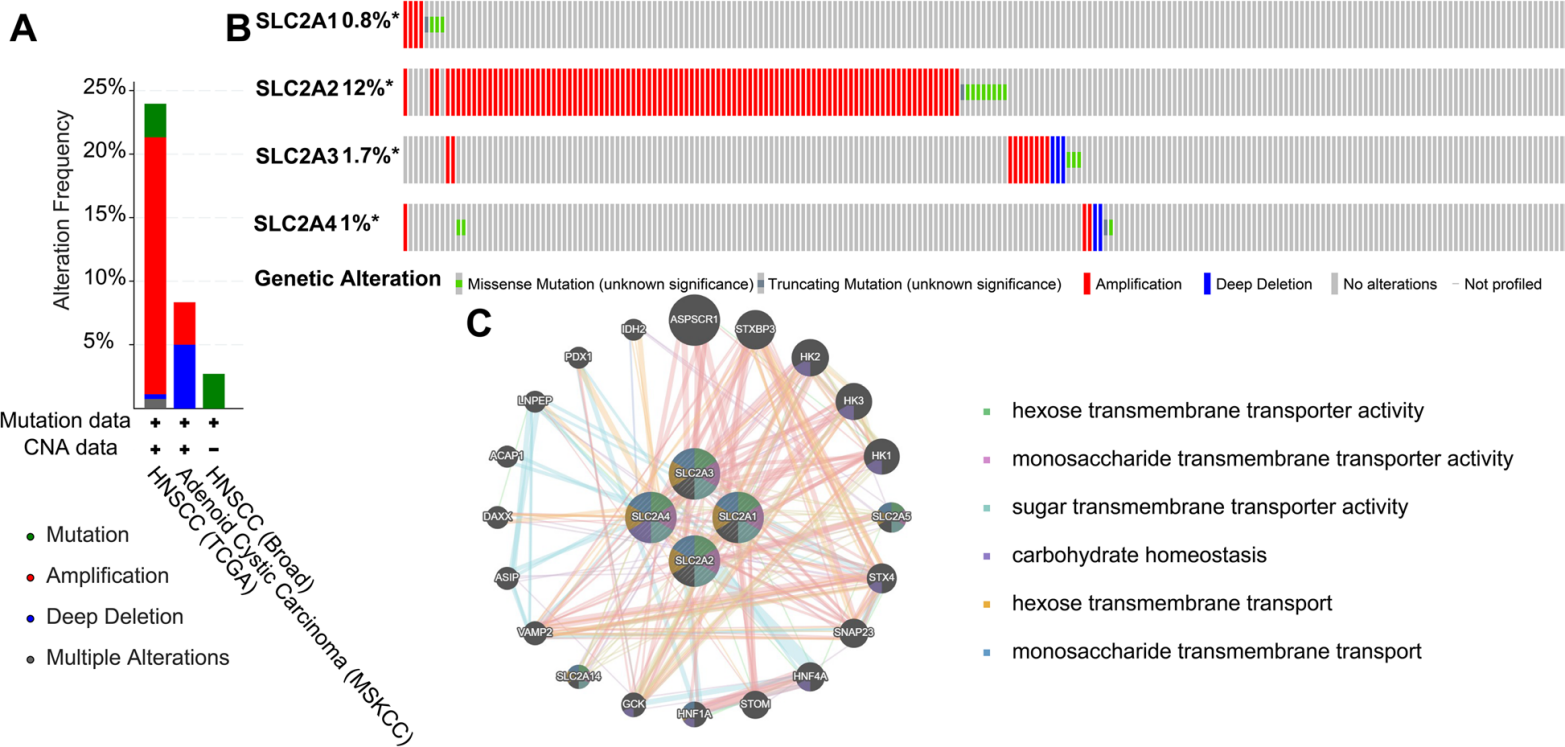
Gene mutation and expression analyses of SLC2A in HNSCC. **A**, **B** Review of alteration changes in differently expressed SLC2As in HNSCC (data from cBioPortal); **C** PPI network of differently expressed SLC2A

Further, we analyzed the genetic alterations of GLUTs in HNSCC patients using the cBioPortal online tool. Many variations were found for different HNSCC subtypes, and amplifications were more frequent in HNSCC samples (Fig. 6A, B). SLC2A1, SLC2A2, SLC2A3, and SLC2A4 were altered in 0.8, 12, 1.7, and 1% of the samples, respectively.

Furthermore, we constructed a PPI network of the differentially expressed SLC2As to analyze their possible interactions. The GeneMANIA results revealed that the functions of differentially expressed SLC2As and their associated molecules (such as ASPSCR1, STXBP3, HK2, HK3, HK1, SLC2A5, and STX4) were primarily related to hexose transmembrane transporter activity, monosaccharide transmembrane transporter activity, sugar transmembrane transporter activity, carbohydrate homeostasis, and hexose transmembrane transport (Fig. 6C).

### Impacts of GLUTs on immune cell infiltration

**Fig. 7:**
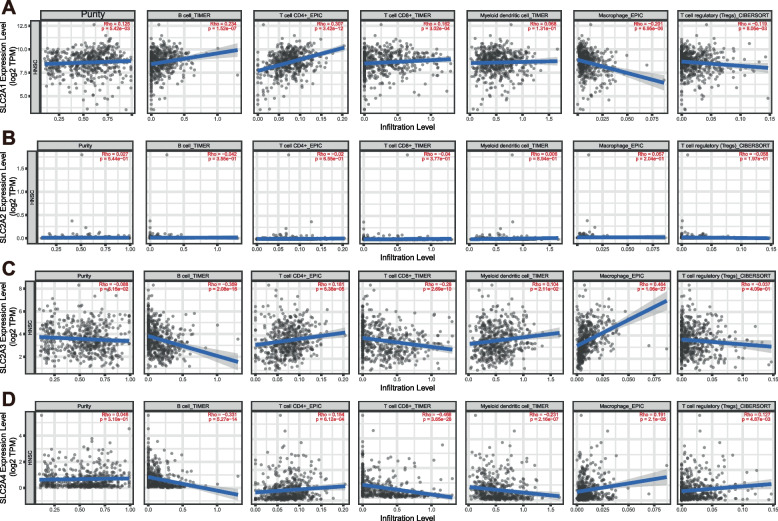
Correlations between the expression of SLC2As and immune cell infiltration (data from TIMER)

Immune cell levels are related to the proliferation and progression of cancer cells, impacting their therapeutic effects. Thus, we used the TIMER database to explore the relationship between SLC2A members and relative immune cell infiltration in HNSCC (Fig. 7). SLC2A1 was connected to the infiltration of B and CD4^+^ T cells but was negatively correlated with macrophages and Treg cells in HNSCC. However, SLC2A2 was not significantly correlated with immune cells in HNSCC patients. SLC2A3 expression was positively related to CD4 + T cells, DCs, and macrophage infiltration. The infiltration of CD4^+^ T cells and macrophages was also related to SLC2A4 expression in HNSCC patients.

### GLUT expression in HNSCC tissues

**Fig. 8:**
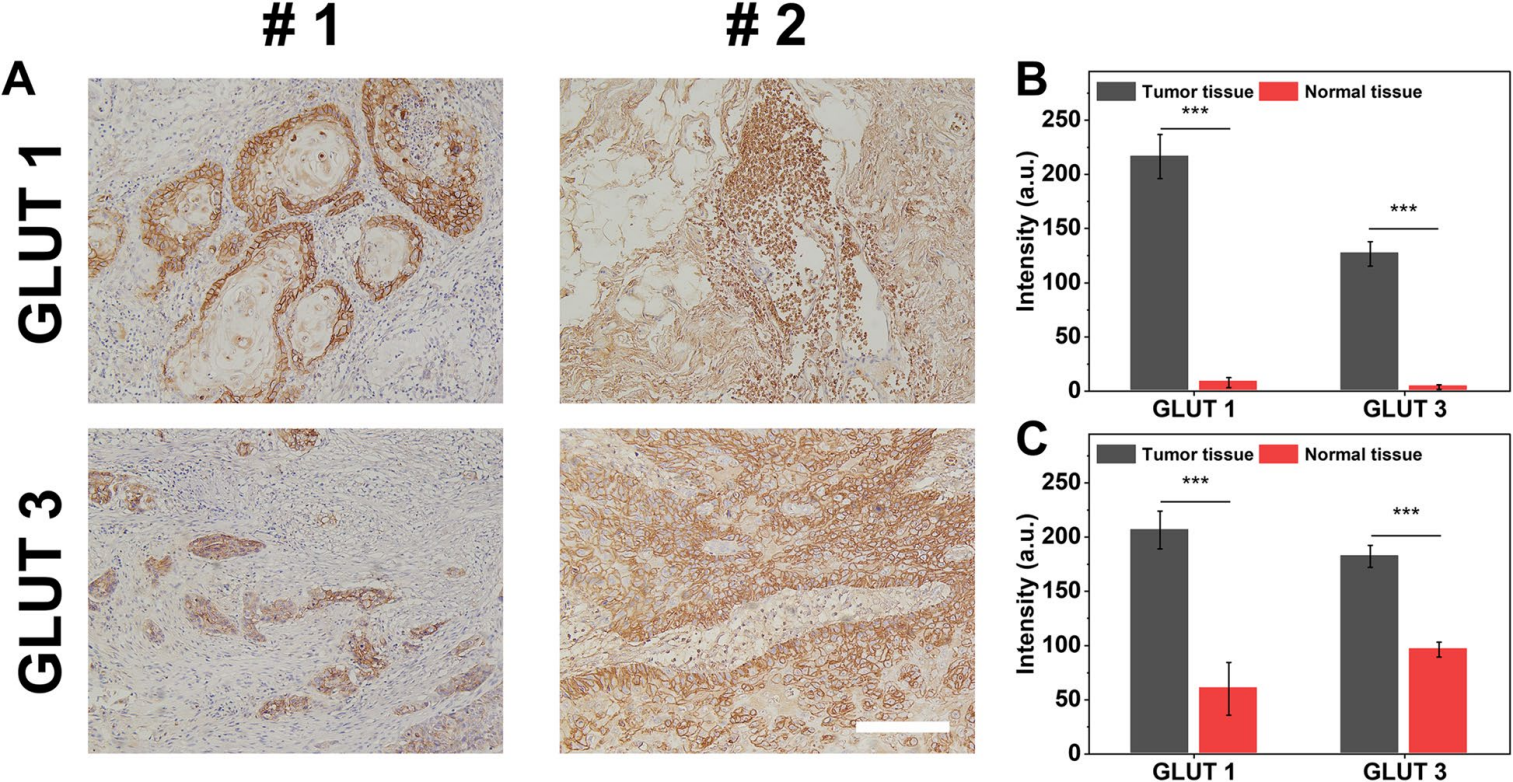
**A** IHC staining of GLUT1 and GLUT3 in HNSCC tissues (Magnification 20x). **B**, **C** Quantitive analysis of (A). ***: *p* < 0.001. Scale bar = 100 μm

To further explore the GLUTs in tumor tissue, we collected samples from HNSCC patients to detect GLUT1 and GLUT3 expression. GLUT 1 and GLUT3 were highly expressed in tumor tissues, consistent with the above results and previous studies (Fig. 8).

## Discussion

HNSCC is common worldwide, and surgery is the primary treatment. Chemotherapy, radiotherapy, and immunotherapy have gradually attracted more attention [18]. It is now widely accepted that HNSCC has no biomarker to test or diagnose early. Thus, patients are usually diagnosed at advanced stages [2], which poses a great difficulty for the treatment and limits its effectiveness. Therefore, a maker for HNSCC is urgently needed.

Many metabolic changes occur in tumor cells, including glycometabolism and lipid metabolism [19]. Glycometabolism is a complicated process, among which the uptake and transport of glucose are vital. GLUT proteins are essential transporters for the uptake and transportation, comprising the beginning of glycometabolism. The primary way for malignant tumor cells to obtain energy is aerobic glycolysis with glucose as substrate. Blood glucose mainly enters the cell through GLUT. The rapid growth of tumor cells needs a high level of glycolysis. It requires more glucose supply and higher levels of GLUT1 expression on the tumor cell surface, increasing glucose uptake and transport. GLUT1 expression is raised in various malignant tumor cells, including liver, pancreas, breast, esophagus, lung, brain, kidney, skin, ovary, colon, endometrial, and cervical malignant tumors, and the positive expression rate reached 50%. GLUT1 can reflect the essential biological characteristics of benign and malignant tumor proliferation and the degree of hypoxia, invasion, and metastasis and objectively evaluate tumor staging, treatment effects, and patient prognoses.

In the epidermal growth factor (EGF)/epidermal growth factor receptor (EGFR) signal transduction pathway related to tumor invasion, GLUT1 is positively correlated with the expression of the EGFR HER-2 and tumor vascular EGF [20]. GLUT1 is directly related to glucose uptake. According to the principle of abnormal glucose metabolism in tumors, GLUT1 might be used as an evaluation index for tumor treatment effects. For example, GLUT1 overexpression is closely related to the types and energy metabolism of various tumors. Cruz et al. performed rectal biopsies for 81 patients undergoing colonoscopy [21]. Significantly increased GLUT1 expression was found in patients with precancerous lesions in the colon. Yan et al. showed that cancer cells from gastric cancer patients overexpressed SLC2A1, which was positively correlated with the proliferation and metastasis of gastric cancer cells [22]. Additionally, high GLUT1 expression is closely related to the occurrence and prognosis of various malignant tumors such as non-small cell lung cancer, prostate cancer, oral squamous cell carcinoma, ovarian cancer, bladder cancer, pancreatic cancer, and melanoma. It has become one of the crucial clinical detection indicators of cell carcinogenesis. Targeting GLUT1 to develop anti-tumor drugs or diagnostic reagents, blocking the source of nutrients for cells, or disguising drugs as nutrients and supplying them to tumor cells in large quantities has become a focus for the development of anti-tumor reagents.

GLUT2, encoded by the SLC2A2 gene, is the major glucose transporter in the liver, pancreas, gut, kidney, and brain, with low affinity and broad substrates such as galactose, mannose, and fructose, in addition to the transport and delivery of glucose. Meanwhile, GLUT2 expression is related to glucose concentration, insulin secretion, the activity of the autonomic nervous system, and regulation of feeding and body temperature. Therefore, GLUT2 is strongly associated with diabetes [23] and autoimmune diabetes. Besides, GLUT2 is highly expressed in some tumors, including liver [24], breast [25], and gastric cancers [26]. In this study, higher GLUT2 expression is associated with lower OS but higher RFS, which may be explained by the possibility that the poorer OS is attained through other factors but not tumor recurrence.

GLUT3 is mainly expressed in nerve cells and platelets. It is also highly expressed in solid tumors such as non-small cell lung cancer, bladder cancer, oral squamous cell carcinoma [11], and laryngeal cancer [27]. GLUT4 is an insulin-sensitive glucose transporter in skeletal, cardiac, and adipose tissue. It mainly regulates glucose absorption and transport to meet the needs of enhanced glucose metabolism. Chang et al. reported that the increased GLUT4 expression was significantly associated with poor OS and RFS in OSCC, which may promote metastasis through the TRIM24/DDX58 axis [28]. Type 2 diabetes, obesity, and aging are also closely related to impaired GLUT4 expression and function [29].

Herein, we evaluated GLUT expression in HNSCC tissues GLUT1 and GLUT3 were highly expressed in the tumor tissue, consistent with the previous results [30]. Also, we found a relationship between the GLUT family and immunity, corresponding to the previous research involving lung adenocarcinoma, prostate cancer, and breast cancer. Based on our findings, the GLUTs family might be used in HNSCC treatments. On the other hand, the detection of immune-related expression was lacking in this study, and we will remedy this limitation in a follow-up study.

## Conclusions

In summary, we analyzed GLUT family members' differential progression and immune infiltration in HNSCC. SLC2A1 and SLC2A3 were significantly increased in HNSCC tissues, while SLC2A4 decreased. SLC2A3 was mainly related to cancer stage development and short OS in HNSCC patients. However, SLC2A1/2 was associated with short OS in HNSCC cancer patients. Furthermore, the expression of GLUTs was significantly related to the infiltration of several immune cells in HNSCC. Our findings might provide a novel strategy to screen a specific prognostic biomarker among GLUTs family members in HNSCC, improving the therapeutic effects in the clinic.

### Supplementary Information

**Additional file 1: Table S1. **The mRNA levels of GLUTs in different types of HNSCC tissues and normal tissues at transcriptome level. 

## Data Availability

All data generated or analyzed during this study are included in this published article. To be noted, in the section ‘Differential mRNA levels’, since ‘Oncomine’ database have been taken offline on 17 January 2022, so the accession numbers could not be checked. Thus related datasets generated and/or analyzed are not publicly available, but are available from the corresponding author on reasonable request.
