# Supplementary material for: Analysis of the relationship between GLUT family in the progression and immune infiltration of head and neck squamous carcinoma
Source: Diagn Pathol. 2023 Aug 4;18:88. doi: 10.1186/s13000-023-01377-x (PMC10401774; doi:10.1186/s13000-023-01377-x)
Supplement: Supplementary file 1 — Additional file 1: Table S1. The mRNA levels of GLUTs in different types of HNSCC tissues and normal tissues at transcriptome level. [file 13000_2023_1377_MOESM1_ESM.docx]

Supporting information

| Name | Types of HNSCC v.s. Nomal tissue | Fold Change | P Value | t-test | Reference or Souce | No |
| --- | --- | --- | --- | --- | --- | --- |
| GLUT1 | Tongue Squamous Cell Carcinoma | 4.612 | 8.20E-8 | 6.663 | BMC Genomics | 38 |
|  | Tongue Carcinoma | 2.154 | 8.11E-6 | 5.060 | Cancer Res 2007/05/15 | 84 |
|  | Head and Neck Squamous Cell Carcinoma | 1.455 | 0.038 | 0.038 | Oncogene 2004/04/01 | 38 |
| GLUT2 | Head and Neck Squamous Cell Carcinoma | 1.274 | 5.22E-52 | 18.686 | TCGA | 628 |
|  | Head and Neck Squamous Cell Carcinoma | 2.497 | 0.027 | 2.523 | Oncogene | 38 |
| GLUT3 | Head and Neck Squamous Cell Carcinoma | 5.590 | 9.57E-17 | 17.130 | Oncogene 2004/04/01 | 38 |
|  | Head and Neck Squamous Cell Carcinoma | 16.709 | 2.39E-23 | 17.315 | Cancer Res 2004/01/01 | 54 |
|  | Thyroid Gland Undifferentiated (Anaplastic) Carcinoma | 1.900 | 0.010 | 3.147 | Clin Cancer Res 2006/04/01 | 99 |
|  | Salivary Gland Adenoid Cystic Carcinoma | 5.004 | 0.050 | 2.003 | Am J Pathol 2002/10/01 | 22 |
|  | Oropharyngeal Carcinoma | 3.317 | 0.006 | 3.532 | Cancer Res 2007/05/15 | 84 |
|  | Oral Cavity Carcinoma | 3.553 | 0.076 | 1.883 | Cancer Res 2007/05/15 84 samples |  |
| GLUT4 | Salivary Gland Adenoid Cystic Carcinoma | 1.174 | 0.099 | 1.344 | Am J Pathol 2002/10/01 22 samples |  |
|  | Hypopharyngeal Squamous Cell Carcinoma | 1.188 | 0.181 | 1.064 | Lab Invest 2005/08/01 12 samples |  |

Table S1. The mRNA levels of GLUTs in different types of HNSCC tissues and normal tissues at transcriptome level.
